# Enthalpy of Formation of Polycyclic Aromatic Hydrocarbons and Heterocyclic Aromatic Compounds

**DOI:** 10.1021/acsomega.5c01887

**Published:** 2025-05-28

**Authors:** Umut Çilesiz, Eren Yaşar Sincer, Burcu Dedeoglu, Viktorya Aviyente

**Affiliations:** † Department of Chemistry, 52949 Bogazici University, Bebek, Istanbul 34342 , Türkiye; ‡ Department of Chemistry, Gebze Technical University, Gebze, Kocaeli 41400, Türkiye

## Abstract

The standard enthalpy of formation is an important indicator of the heat involved in a chemical reaction. In this work, benchmark calculations with quasi-isodesmic type reactions have been performed on 8 different polycyclic aromatic hydrocarbons (PAHs) with 9 different methodologies. All geometry optimizations were carried out at the B2PLYP-D3, B3LYP-D3, CAM-B3LYP-D3, LC-WPBE-D3, M05-2X-D3, M06-2X-D3, WB97XD, DSDPBEP86, and PBE0DH levels in conjunction with the cc-pVTZ basis set. The DSDPBEP86-optimized isodesmic reactions yield remarkably good agreement with the experimental data for most of the compounds. For the heterocyclic aromatic compounds, quasi-isodesmic reactions are carried out successfully using the cost-effective B2PLYP-D3/cc-pVTZ and B3LYP-D3/cc-pVTZ methodologies. In the case of alkyl-substituted thiophene derivatives, quasi-isodesmic reactions and the connectivity-based hierarchy (CBH) methods have yielded enthalpies of formation close to those from experiments with B2PLYP-D3/cc-pVTZ.

## Graphical abstract

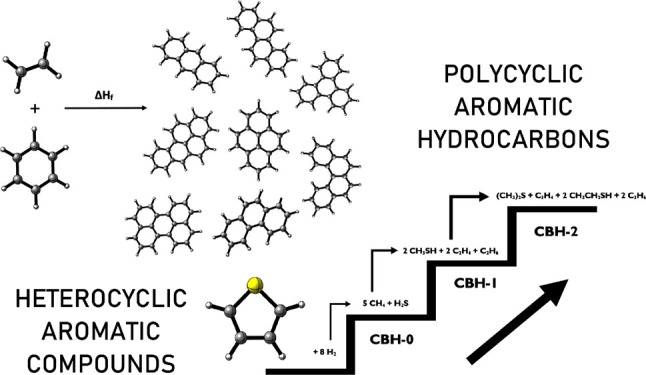

## Introduction

Thermochemical data play a significant role in chemistry as they allow chemists to predict the thermodynamic properties of reactions. The enthalpy of formation, which is the change in enthalpy when one mole of a substance is formed from its elements in standard states, is a thermochemical property utilized in calculating reaction enthalpies and provides insight into the stability of the resultant molecule. In recent years, computational methods for calculating the enthalpy of formation have gained significant popularity, especially in the absence of experimental thermochemical data.^1^

Polycyclic aromatic hydrocarbons (PAHs) are composed of carbon and hydrogen atoms that form two or more fused aromatic rings. They are produced and released during the combustion of fuels.^2–4^ Regarding the risks to health and the environment caused by PAHs and considering that the primary source of PAH molecules is combustion from human activities, it is essential to cultivate a comprehensive understanding of their chemistry. PAHs have also gained popularity in theoretical and experimental studies due to their applications in nanostructured materials such as fullerenes and graphene.^5,6^

In 2008, Roux and Temprado published an extensive report on experimental thermochemical data for 63 polycyclic aromatic hydrocarbons.^7^ This study critically evaluated enthalpies of formation in the condensed state along with sublimation, vaporization, and fusion enthalpies, referencing over 350 articles. In 2015, Allison and Burgess conducted a study on 669 PAH molecules to predict the enthalpy of formation for these molecules.^1^ The authors extrapolated the B3LYP-D3/cc-pVDZ results to a larger basis set limit and applied a group-based approach, resulting in a mean unsigned deviation of 5.0 kJ/mol and a root-mean-square deviation of 6.4 kJ/mol from the experimental data. In 2021, Karton and Chan evaluated the enthalpy of formation of 20 PAHs with the explicitly correlated W1–F12 thermochemical procedure via atomization reactions and quasi-isodesmic reactions. They found that as the size of the molecule increases, the differences in calculated enthalpy of formation values from various methods also increase.^8^ In 2022, Dorofeeva and Andreychev investigated the enthalpy of formation for 30 PAHs using the DLPNO–CCSD­(T1)/CBS method.^9^ Their study emphasized the accuracy and reliability of modern quantum chemical methods, particularly in cases where experimental data is lacking. The same year, Xu et al. utilized the connectivity-based hierarchy (CBH) method to obtain standard enthalpy of formation values for 50 PAHs, demonstrating that CBH is an efficient and precise approach.^10^ Very recently, efficient reaction-based approaches for gas-phase enthalpy of formation prediction and their application to large (C32) polycyclic aromatic hydrocarbons have been reported.^11^

Heterocyclic aromatic compounds (HACs) are cyclic hydrocarbons in which one or more carbon atoms are replaced by heteroatoms. Similar to polycyclic aromatic hydrocarbons (PAHs), HACs can be produced as byproducts from the combustion of petroleum or coal.^12,13^ Thiophene, a five-membered aromatic cyclic compound containing sulfur, and its derivatives have garnered attention over the years due to their applications in modern drug design, biochemistry, as well as electronic and optoelectronic devices.^14,15^ Ribeiro Da Silva et al. conducted both experimental and computational studies on the thermochemical properties of thiophene and its derivatives.^16–18^ In 2011, Zauer calculated the enthalpy of formation for 21 carbonyl derivatives of thiophene in the gas phase, emphasizing that the PM3 method provided the best correlation with experimental data compared to other methods.^19^ In 2015, Nikoofard reported a computational study on the enthalpy of formation of β-alkylthiophenes and concluded that alkyl-substituted thiophenes exhibit favorable characteristics as conducting polymers.^14^

Pyridine is widely utilized across various fields, including pharmaceuticals, polymers, agriculture, and organocatalysis.^20,21^ Ribeiro Da Silva et al. experimentally determined the standard molar enthalpy of formation for 2,4,6-trimethylpyridine and several bipyridines.^22,23^ Zauer computed the heat of formation for 63 nitrogen-containing cyclic compounds using the PM3 method, demonstrating a strong correlation with experimental data.^24^ Ramabhadran and Raghavachari applied the CBH method to accurately predict the thermochemical properties of both hydrocarbons and nonhydrocarbons.^25^ However, the CBH method is not recommended for molecules that exhibit aromaticity and ring strain due to the presence of potential resonance structures. In their study, geometry optimizations were carried out at the B3LYP/6–31G­(2df,p) level, and single-point calculations were performed by using the HF, MP2, and CCSD­(T) methods. The findings indicated that, for relatively large nonaromatic molecules, the results obtained with the MP2 method at the CBH-2 rung are comparable to those achieved using the more expensive CCSD­(T) method at the CBH-3 rung.^24^

In this study, we selected eight polycyclic aromatic hydrocarbon molecules: anthracene, phenanthrene, pyrene, chrysene, benzo­[c]­phenanthrene, triphenylene, perylene, and benzopyrene, as well as thiophene and pyridine, along with their derivatives, to calculate their gas phase enthalpy of formation (Figure 1). For the first time, we assessed the accuracy of cost-efficient computational methods for heterocyclic aromatic hydrocarbons. We applied a promising connectivity-based hierarchical method to alkyl-substituted heterocyclic aromatic hydrocarbons, where the use of isodesmic reactions proves to be less effective.

**Figure 1:**
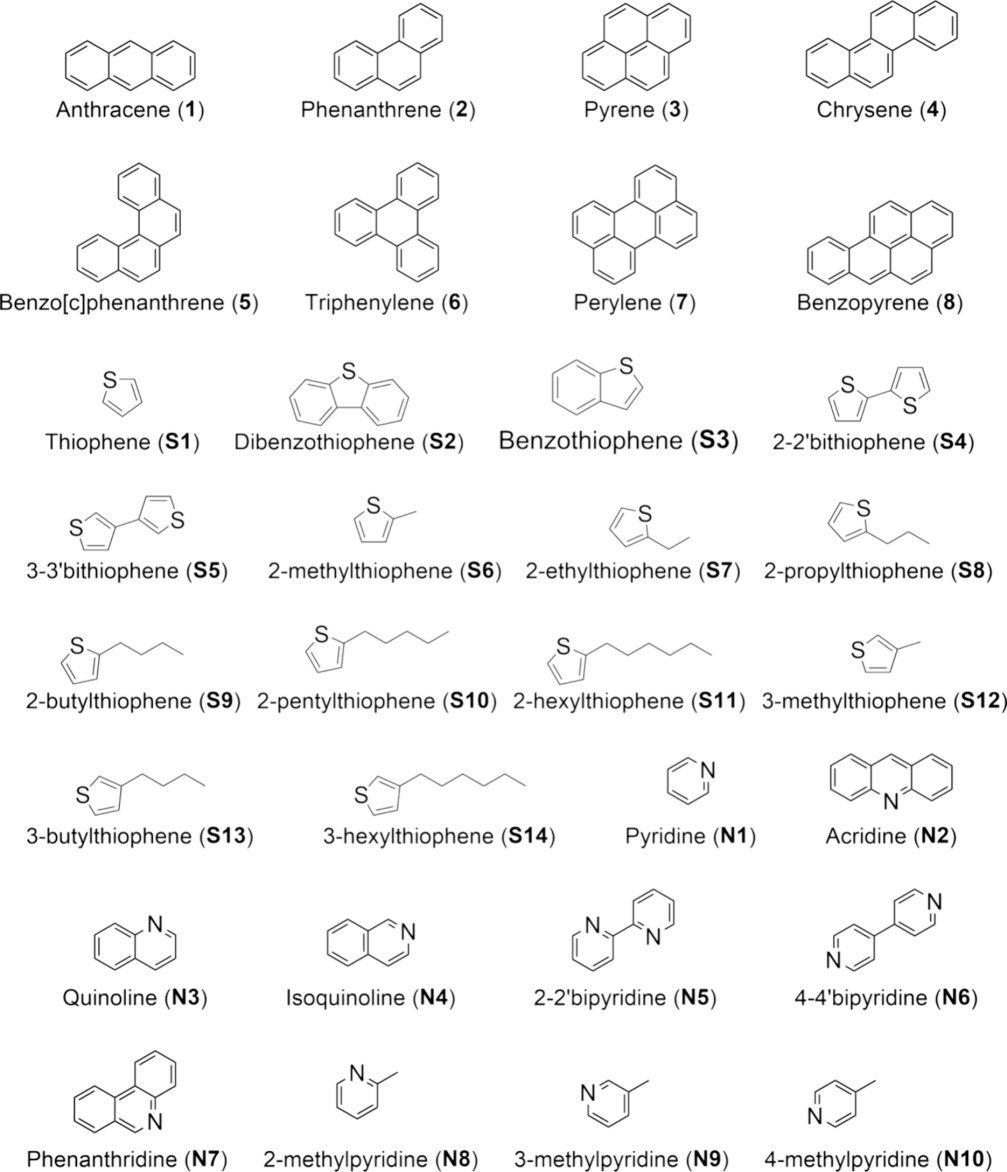
Polycyclic aromatic hydrocarbons (PAH) and heterocyclic aromatic compounds (HAC) considered in this study.

## Computational Details

### Polycyclic Aromatic Hydrocarbons

In order to compare the performance of various DFT functionals on the prediction of the enthalpy of formation energies, a comprehensive literature search was carried out. In 2017, Karton compared the performance of 49 DFT functionals among the rungs of Jacob’s Ladder to predict the relative energies of polycyclic aromatic hydrocarbon isomers.^26^ They concluded that the generalized gradient approximation and meta-generalized gradient approximation functionals underestimate the isomerization energies. They suggested CAM-B3LYP to be the best method to choose among the range-separated DFT functionals, and the double-hybrid DFT functionals yielded the best performance for the estimation of the isomerization energies. In 2021, Xu et al. tested the M06-2X, wB97XD, and B2PLYP-D3 methods for bond dissociation energies and the enthalpy of formation for chlorinated and brominated PAHs.^27^ They concluded that the wB97xD functional performed the best for their study of thermodynamic and kinetic properties.

After careful investigation of the thermochemical and kinetic studies of the PAHs, we have decided to test 6 different functionals from the fourth rung of the Jacob’s Ladder (B3LYP,^28^ CAM-B3LYP,^29^ LC-WPBE,^30^ M05-2X-D3 ^31,32^ M06-2X-D3^32^ and WB97XD^33^ and three different functionals from the fifth rung of the Jacob’s Ladder (B2PLYP,^34^ DSDPBEP86,^35^ PBE0DH^36^). The functionals B3LYP, CAM-B3LYP, LC-WPBE, M05-2X, M06-2X ,^32^ and B2PLYP with empirical D3 ^37^ corrections have been utilized. We used the cc-pVTZ basis set in conjunction with the above-mentioned functionals. All the geometry optimizations were carried out with the functionals mentioned above as implemented in Gaussian 16.^38^ (Table 1).

**Table 1:** Experimental and Calculated Enthalpy of Formation (kcal/mol, 298 K), Δ*H*_f_ Values for Polycyclic Aromatic Hydrocarbons, and Absolute Differences

	1	(Δ*H*_f_)_Exp_ – 54.9 ^7^	2	(Δ*H*_f_)_Exp_ – 48 ^7^
	Δ*H*_f_	|exp – calc|	Δ*H*_f_	|exp – calc|
B3LYP-D3	56.5	1.7	51.5	3.2
CAM-B3LYP-D3	58.6	3.8	52.4	4.1
LC-WPBE-D3	59.6	4.8	52.5	4.2
M05-2X-D3	59.2	4.4	52.8	4.5
M06-2X-D3	58.7	3.9	52.3	4.0
WB97XD	59.4	4.6	53.1	4.8
B2PLYP-D3	55.9	1.1	50.4	2.1
DSDPBEP86	54.8	0.0	49.1	0.8
PBE0DH	59.2	4.3	53.3	4.9

	3	(Δ*H*_f_)_Exp_ – 54.0 ^7^	4	(Δ*H*_f_)_Exp_ – 64.0 ^7^
	Δ*H*_f_	|exp – calc|	Δ*H*_f_	|exp – calc|
B3LYP-D3	57.3	3.4	68.0	3.8
CAM-B3LYP-D3	59.4	5.5	69.5	5.3
LC-WPBE-D3	59.8	5.9	69.7	5.5
M05-2X-D3	59.7	5.8	70.0	5.8
M06-2X-D3	59.3	5.4	69.3	5.1
WB97XD	60.3	6.4	70.4	6.2
B2PLYP-D3	55.6	1.7	66.1	1.9
DSDPBEP86	53.9	0.0	64.2	0.2
PBE0DH	59.6	5.7	70.6	6.4

	5	(Δ*H*_f_)_Exp_ – 69.7 ^7^	6	(Δ*H*_f_)_Exp_ – 64.6 ^7^
	Δ*H*_f_	|exp – calc|	ΔH_f_	|exp – calc|
B3LYP-D3	73.8	4.2	68.6	4.0
CAM-B3LYP-D3	75.5	5.9	69.3	4.7
LC-WPBE-D3	75.5	5.9	68.7	4.1
M05-2X-D3	75.7	6.1	69.8	5.2
M06-2X-D3	75.0	5.4	68.9	4.3
WB97XD	76.3	6.7	68.9	4.3
B2PLYP-D3	71.8	2.2	66.2	1.6
DSDPBEP86	69.6	0.1	63.7	0.9
PBE0DH	76.7	7.0	70.6	6.0

	7	(Δ*H*_f_)_Exp_ – 76.1 ^7^	8	(Δ*H*_f_)_Exp_ – 72.2 ^7^
	Δ*H*_f_	|exp – calc|	Δ*H*_f_	|exp – calc|
B3LYP-D3	80.5	4.4	75.5	3.4
CAM-B3LYP-D3	83.0	6.9	78.3	6.2
LC-WPBE-D3	83.1	7.0	78.9	6.8
M052X-D3	83.6	7.5	78.9	6.8
M062X-D3	82.7	6.6	78.2	6.1
WB97XD	83.2	7.1	79.5	7.4
B2PLYP-D3	77.9	1.8	73.0	0.9
DSDPBEP86	75.2	0.9	70.5	1.8
PBE0DH	83.8	7.7	78.8	6.6

For the gas phase enthalpy of formation calculations, the quasi-isodesmic eq 1 is used, with the corresponding coefficients *a* and *b* reported in Table S1.

$$C_{n}H_{m}\to aC_{6}H_{6}+bC_{2}H_{4}$$ 1

### Heterocyclic Aromatic Compounds

In 1970, Hehre and coworkers contributed to the literature with a study concerning the accuracy of the theoretical prediction of the thermochemical properties of organic molecules with isodesmic reactions.^39^ In isodesmic reactions, bond types between the heavy atoms in the molecules are conserved. However, this method is not quite applicable and reliable for large molecules containing multiple aromatic rings, because of the large errors in the calculated enthalpy of formation values. To overcome this problem, in 2005, Sivaramakrishnan et al. proposed ring-conserved isodesmic reactions, which aim to conserve the ring fragments as well as bond types.^40^ The isodesmic reactions used for the calculation of the enthalpy of formation of the heterocyclic aromatic compounds are reported in Figures 2 and 3.

**Figure 2:**
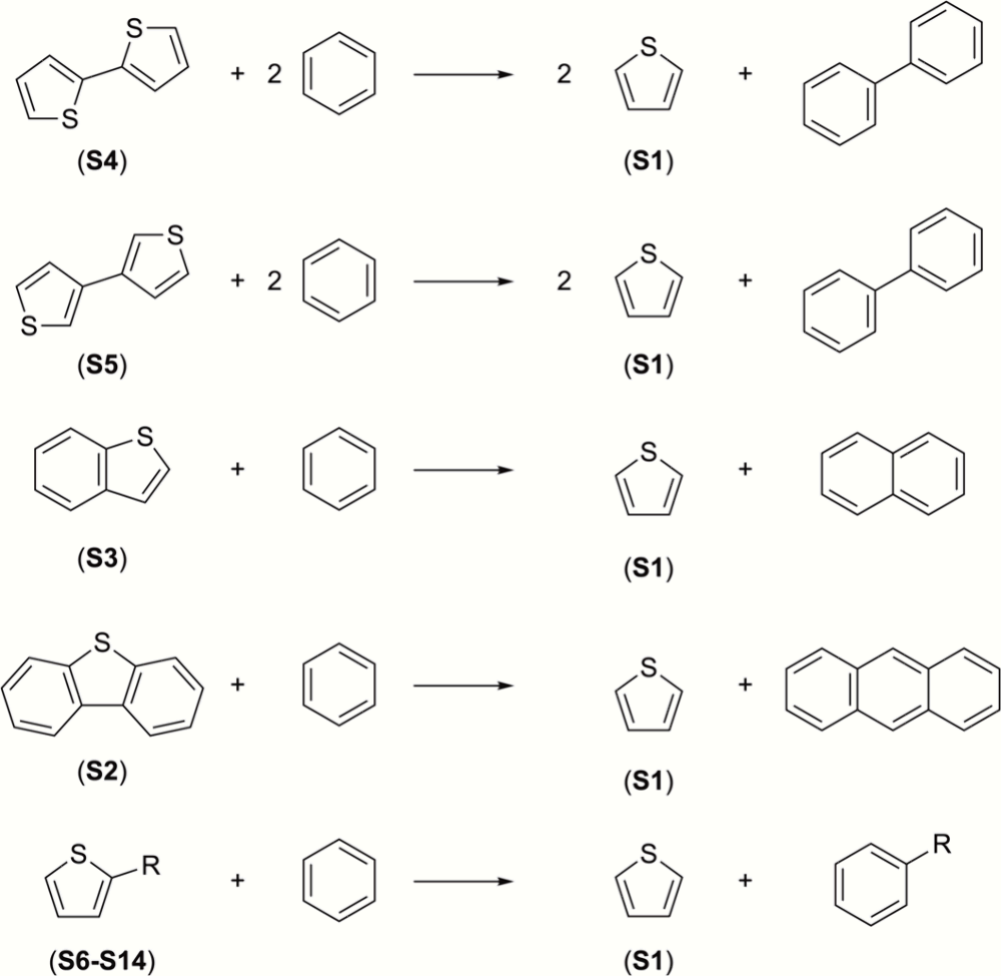
Isodesmic reactions of thiophene derivatives (**S6** = 2-methylthiophene; **S7** = 2-ethylthiophene; **S8** = 2-propylthiophene; **S9** = 2-butylthiophene, **S10** = 2-pentylthiophene; **S11** = 2-hexylthiophene); **S12** = 3-methylthiophene; **S13** = 3–butylthiophene; **S14** = 3-hexylthiophene.

**Figure 3:**
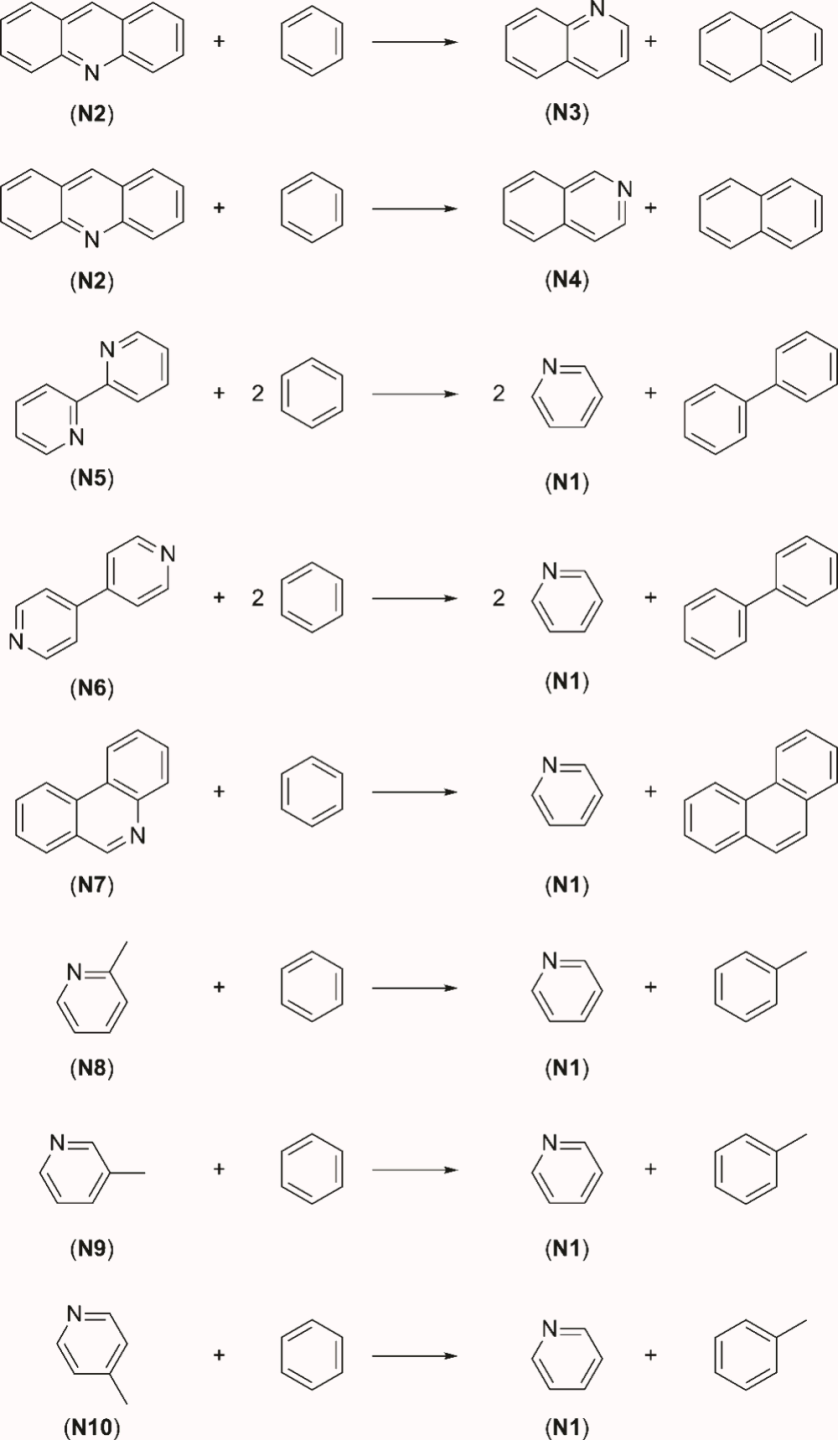
Isodesmic reactions of the pyridine derivatives.

The experimental values of the heterocyclic aromatic compounds, except for thiophene, dibenzothiophene, 2–2’-bithiophene, and 3–3′-bithiophene, were retrieved from the NIST Chemistry WebBook.^41^ The enthalpy of formation values of thiophene and dibenzothiophene were taken from Thermochemical Data of Organic Compounds by Pedley et al.,^42^ while the values of 2–2’-bithiophene and 3–3′bithiophene are from the study of Ribeiro da Silva et al.^16^ Among the alkyl-substituted thiophenes, experimental values for 2-methyl thiophene and 3-methyl thiophene were retrieved from NIST, and the rest were gathered from the study of Ribeiro da Silva et al.^18^

### Alkyl-Substituted Thiophene Derivatives

For the alkyl substituted HACs, a more accurate method, the connectivity-based hierarchy (CBH) is applied, as well as isodesmic reaction calculations. In 2011, Ramabhadran and Raghavachari developed this theoretical method to predict the reaction energy for closed-shell organic molecules.^43^

CBH is a simple and efficient method that allows chemists to create reaction schemes, protecting the connectivity of atoms without a need for predefined reactants. This method requires only knowledge of the structural formula of the molecule. Starting from the zeroth rung (the so-called isogyric scheme), reactions are constructed with the expansion of the protected atoms or bonds around the atoms in every rung. In the zeroth rung, only the heavy atoms in the organic molecules are extracted and saturated with enough number of hydrogens, and the reaction is balanced by adding the necessary number of H_2_ molecules to the reactants. Moving to the first rung, the number of covalent bond types between the heavy atoms in the organic molecules is conserved, and the reaction scheme is completed by adding the products of the zeroth rung as the reactants of the first rung. CBH first rung reaction scheme is based on the isodesmic bond separation scheme. In the second rung, the immediate bonding environment of every heavy atom in an organic molecule is preserved. Again, the reaction scheme is constructed by using the product of the first rung as the reactants of the second rung, with the fragments extracted preserving the bonding environment, which is equivalent to the hypohomodesmotic reaction scheme. More complex reaction schemes for the upper rungs can be constructed simply by following the same steps within every rung, expanding the atom or bonding environment.^43^

## Results and Discussion

### Polycyclic Aromatic Hydrocarbons

The calculated heats of formation together with the experimental enthalpy of formation values of the PAH molecules from the study of Roux et al.^7^ are gathered in Table 1. In most cases, the DSDPBEP86 methodology performed the best with the lowest deviations from the experimental values; the absolute errors ranging from 0 to 1.8 kcal/mol with a mean unsigned error (MUE) of 0.6 kcal/mol. The other MP2-based B2PLYP-D3/cc-pVTZ methodology ranks second in the evaluation of the enthalpy of formation, yielding absolute errors from 0.9 to 2.2 kcal/mol (MUE = 1.7 kcal/mol). In the calculations performed for PAH molecules in this study, the B2PLYP-D3 method has shown moderate computational time as compared to DSDPBEP86 due to the spin-component-scaled treatment of MP2 in the latter as opposed to the normal MP2 treatment in B2PLYP-D3. Among the hybrid functionals, B3LYP-D3 performs better than the others with absolute errors 1.7–4.4 kcal/mol (MUE = 3.5 kcal/mol). With the other functionals, the absolute differences typically range between 3.8 and 7.5 kcal/mol, not favoring one over the others (Tables 1 and 2).

**Table 2:** Mean Unsigned Errors (MUE, kcal/mol) of Different DFT Methods

	(MUE)
B3LYP-D3	3.5
CAM-B3LYP-D3	5.3
LC-WPBE-D3	5.5
M05-2X-D3	5.8
M06-2X-D3	5.1
WB97XD	5.9
B2PLYP-D3	1.7
DSDPBEP86	0.6
PBE0DH	6.1

### Heterocyclic Aromatic Compounds

For the calculation of the enthalpy of formation of heterocyclic aromatic compounds, B2PLYP-D3 is chosen among the double-hybrid functionals based on its good general performance and moderate computational time. In the study based on the evaluation of heats of formation of medium-sized organic compounds performed by Minenkov et al., B2PLYP-D3 has yielded reasonable MUEs (<4 kcal/mol).^44^ For comparison purposes, the hybrid functional B3LYP-D3, known for its accuracy in reproducing geometries and its balance between computational cost and accuracy, has also been used.

The enthalpies of formation of thiophene, pyridine, and their derivatives were evaluated using the isodesmic reactions (Table 3). Note that the enthalpies of formation of **S2**, **S3**, **S4**, and **S5** are calculated by using the experimental value for thiophene (**S1**). The alkyl-substituted thiophene derivatives (**S6**–**S14**) benefit from the experimental heat of formation value of **S1** as well as those of the alkyl-substituted benzene derivatives. As the carbon chain attached to thiophene gets longer, so does the deviation from the experimental value. B2PLYP-D3/cc-pVTZ gives slightly better agreement with experiment than the B3LYP-D3/cc-pVTZ methodology. Note that the largest deviations from the experiment are for **S9, S10**, and **S11.** For these molecules, the long-range interactions between the hydrogen atoms of the alkyl chain and the sulfur atom on the thiophene ring may not have been accounted for in the quasi-isodesmic reaction: the right-hand sides of the equations do not display any S–H interactions as opposed to the left-hand sides.

**Table 3:** Experimental and Calculated Enthalpies of Formation (kcal/mol, 298 K) of Thiophene Derivatives and Pyridine Derivatives with B3LYP-D3/cc-pVTZ and B2PLYP-D3/cc-pVTZ Using Isodesmic Reactions

		B3LYP-D3/cc-pVTZ		B2PLYP-D3/cc-pVTZ	
	(Δ*H*_f_)_Exp_	Δ*H*_f_	|exp – calc|	Δ*H*_f_	|exp – calc|
**S1**	27.5 ^42^	28.4	0.9	27.5	0.0
**S2**	49.0 ^42^	49.9	0.9	50.4	1.4
**S3**	39.8 ^41^	39.9	0.2	40.3	0.6
**S4**	59.2 ^16^	57.3	1.9	59.1	0.1
**S5**	59.2 ^16^	58.2	1.0	58.5	0.7
**S6**	20.2 ^41^	19.5	0.7	20.1	0.1
**S7**	12.7 ^18^	14.6	1.8	14.4	1.7
**S8**	7.8 ^18^	9.3	1.5	9.1	1.3
**S9**	1.9 ^18^	4.4	2.5	4.2	2.3
**S10**	–3.1 ^18^	–0.4	2.7	–0.5	2.6
**S11**	–7.6 ^18^	–5.4	2.2	–5.5	2.1
**S12**	19.7 ^41^	19.5	0.3	20.1	0.4
**S13**	3.7 ^18^	4.7	1.0	4.4	0.8
**S14**	–6.9 ^18^	–5.0	1.9	–5.3	1.6
**N1**	33.5 ^41^	33.0	0.5	32.9	0.6
**N2**	65.5 ^41^	66.3	0.8	66.3	0.8
**N3**	47.9 ^41^	47.1	0.8	47.1	0.8
**N4**	48.9 ^41^	48.2	0.7	48.3	0.7
**N5**	64.0 ^41^	65.1	1.0	65.2	1.1
**N6**	70.1 ^41^	70.0	0.0	70.2	0.1
**N7**	57.5 ^41^	60.6	2.6	59.9	2.4
**N8**	23.7 ^41^	23.5	0.2	23.6	0.2
**N9**	24.8 ^41^	25.3	0.5	25.3	0.5
**N10**	24.8 ^41^	24.7	0.1	24.7	0.1

The isodesmic reactions for **N2**, **N3**, and **N4** include the heats of formation of benzene and naphthalene and are correlated to each other. **N5**–**N10** are expressed in terms of benzene, pyridine, and the corresponding aromatic molecules. Among all the molecules, **N7**, namely phenanthridine, stands out as an outlier with the highest deviation from experiment.

### Alkyl-Substituted Thiophene Derivatives

For alkyl-substituted thiophene derivatives, where long-range interactions between heteroatoms and hydrogen atoms may not be well accounted for in quasi-isodesmic reactions, the Connectivity Based Hierarchy (CBH) is also applied, as described in the methodology part. The results obtained with both methods are compared with the experimental values taken from the study of Ribeiro da Silva et al.,^18^ and all the other experimental values of the compounds used in CBH reactions were retrieved from the NIST Chemistry WebBook.^41^ As in the previous section, calculations have been performed with the B3LYP-D3 and B2PLYP-D3 levels of theory and the cc-pVTZ basis set.

Comparison of the calculated enthalpy of formation values for alkyl-substituted thiophenes with the experimental values is given in Table 4. The double hybrid B2PLYP-D3/cc-pVTZ performs slightly better, with absolute errors ranging from 0.1 to 2.6 kcal/mol, compared to B3LYP-D3/cc-pVTZ, which yields absolute errors between 0.7 and 2.7 kcal/mol. A further improvement is observed when the CBH method is applied using B2PLYP-D3/cc-pVTZ, where the absolute errors lie between 0.0 and 2.1 kcal/mol. Note that the heats of formation of **S6** (2-methylthiophene) and **S12** (3-methylthiophene) are reproduced almost exactly. Since these molecules are small, they show similar behavior on both sides of the quasi-isodesmic equation.

**Table 4:** Comparison of the Calculated Enthalpy of Formation of Thiophene and Its Alkyl Derivatives with Isodesmic Reactions and the CBH Method Using B3LYP-D3/cc-pVTZ and B2PLYP-D3/cc-pVTZ (kcal/mol, 298 K)

		B3LYP-D3/cc-pVTZ				B2PLYP-D3/cc-pVTZ			
		CBH		isodesmic		CBH		isodesmic	
	(Δ*H*_f_)_Exp_	Δ*H*_f_	|exp – calc|	Δ*H*_f_	|exp – calc|	Δ*H*_f_	|exp – calc|	Δ*H*_f_	|exp – calc|
**S6**	20.2 ^41^	20.4	0.3	19.5	0.7	19.7	0.4	20.1	0.1
**S7**	12.7 ^18^	17.0	4.3	14.6	1.8	14.2	1.5	14.4	1.7
**S8**	7.8 ^18^	11.8	4.0	9.3	1.5	9.0	1.2	9.1	1.3
**S9**	1.9 ^18^	6.8	4.9	4.4	2.5	4.0	2.1	4.2	2.3
**S10**	–3.1 ^18^	1.9	4.9	–0.4	2.7	–1.0	2.1	–0.5	2.6
**S11**	–7.6 ^18^	–3.1	4.5	–5.4	2.2	–5.9	1.6	–5.5	2.1
**S12**	19.7 ^41^	20.4	0.7	19.5	0.3	19.7	0.0	20.1	0.4
**S13**	3.7 ^18^	7.1	3.5	4.7	1.0	4.3	0.6	4.4	0.8
**S14**	–6.9 ^18^	–2.7	4.1	–5.0	1.9	–5.6	1.3	–5.3	1.6

#### Construction of the Zeroth, First, and Second Rung Reaction Schemes for Thiophene Derivatives

The zeroth-rung (CBH-0) reaction scheme for methylthiophene is constructed by preserving the heavy atom number and balancing the equation with enough H_2_ molecules (Scheme 1). In the first rung (CBH-1), covalent bonds are conserved between the heavy atoms in the organic molecule, giving 2 methanethiols, 2 ethenes, and 2 ethanes as products. In the second rung (CBH-2), the immediate bonding environments as well as the atoms on the heavy atoms in the organic molecule are conserved, resulting in dimethylsulfide, 2-ethenethiol, and 2-propene. Note that there is no branching point or terminal atoms in this molecule; for molecules containing these structural properties, reaction schemes are constructed accordingly.

**Scheme 1:**
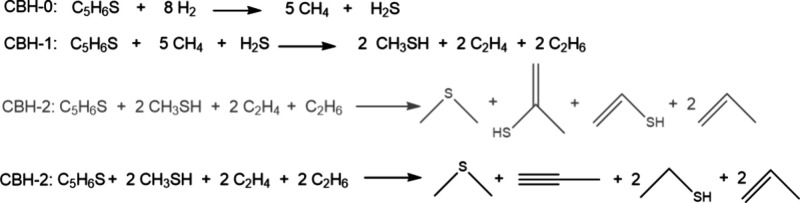
CBH-0, CBH-1, and CBH-2 Reaction Schemes for Methyl Thiophene^a^

a The standard reaction of CBH-2 (transparent) could not be used due to the lack of experimental data.

In the reaction scheme of the last rung (CBH-2), because of the lack of experimental data of the enthalpy of formation of ethenethiol, the reaction involving ethenethiol as the product is omitted (transparent in Scheme 1) but instead migration of two carbon–carbon double bonds from the ethenethiol to propene is carried out resulting in 2 ethanethiol and 2 propyne molecules. When the adjustment is needed, appropriate bond migrations are carried out. In this study, we have built the reactions until the second rung (CBH-2) and proceeded to calculate the reaction energy from there on. Schemes for CBH calculations (**S6**–**S14**) are displayed in Figure S1.

## Conclusions

In this study, benchmark calculations with quasi-isodesmic type of reactions have been performed on 8 different polycyclic aromatic hydrocarbons (PAHs) with 9 different methodologies. Optimizations at B3LYP-D3, CAM-B3LYP-D3, LC-WPBE-D3, M05–2X-D3, M06–2X-D3, WB97XD, B2PLYP-D3, DSDPBEP86, and PBE0DH levels with the cc-pVTZ basis set are performed. In most cases, the analysis of the results indicates that DSDPBEP86 yielded results closer to the experimental values as compared to the other methods. The B3LYP-D3/cc-pVTZ and B2PLYP-D3/cc-pVTZ methodologies have been used for the calculation of the enthalpy of formation of thiophene, pyridine, and their derivatives. The isodesmic reactions, as well as the CBH method, until the second rung, are also used for the evaluation of the enthalpy of formation of alkyl-substituted thiophene derivatives. For the compounds tested, the CBH method with B2PLYP-D3/cc-pVTZ yielded comparable results to the experiment. Future research should focus on expanding the list of compounds and developing more accurate and computationally efficient methods for predicting the enthalpy of formation of HACs, incorporating advanced quantum chemical techniques and machine learning algorithms to enhance the reliability of theoretical predictions in the absence of experimental data.

## Supplementary Material



## Notes

Coefficients of benzene and ethylene for the isodesmic reactions used in the calculation of the enthalpy of formation for the corresponding molecules; zeroth, first, and second rung CBH reactions of alkyl-substituted thiophenes (PDF)
