# Supplementary material for: Enthalpy of Formation of Polycyclic Aromatic Hydrocarbons and Heterocyclic Aromatic Compounds
Source: ACS Omega. 2025 May 28;10(22):23433–9. doi: 10.1021/acsomega.5c01887 (PMC12163763; doi:10.1021/acsomega.5c01887)
Supplement: Supplementary file 1 [file ao5c01887_si_001.pdf]

Supporting Information

Enthalpy of Formation of Polycyclic Aromatic Hydrocarbons and Heterocyclic Aromatic Compounds

Umut Çilesiz,[a] Eren Yaşar Sincer,[a] Burcu Dedeoğlu,[b] Viktorya Aviyente*[a]

[a] Department of Chemistry, Bogazici University, Istanbul, 34342 Bebek, Turkey

[b] Department of Chemistry, Gebze Technical University, Gebze, Kocaeli, 41400, Turkey

Table S1. Coefficients of benzene and ethylene for the isodesmic reactions used in the calculation of the enthalpy of formation for corresponding molecules.

C_nH_m	Molecule	(a)	(b)
$C_{14}H_{10}$	anthracene	3	-2
$C_{14}H_{10}$	phenanthrene	3	-2
$C_{16}H_{10}$	pyrene	3 2/3	-3
$C_{18}H_{12}$	chrysene	4	-3
$C_{18}H_{12}$	benzo[c]phenanthrene	4	-3
$C_{18}H_{12}$	triphenylene	4	-3
$C_{20}H_{12}$	perylene	14/3	-4
$C_{20}H_{12}$	benzopyrene	14/3	-4

2-methylthiophene – S6

CBH-0: $C_5H_6S + 8\ H_2 \longrightarrow 5\ CH_4 + H_2S$

CBH-1: $C_5H_6S + 5\ CH_4 + H_2S \longrightarrow 2\ CH_3SH + 2\ C_2H_4 + 2\ C_2H_6$

CBH-2: $C_5H_6S + 2\ CH_3SH + 2\ C_2H_4 + C_2H_6 \longrightarrow$ 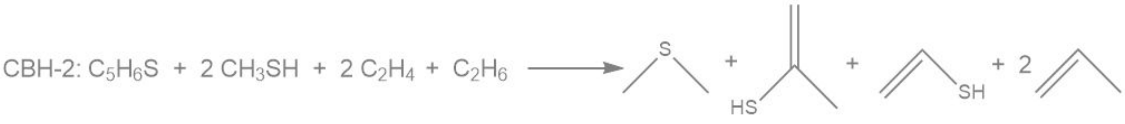

CBH-2: $C_5H_6S + 2\ CH_3SH + 2\ C_2H_4 + 2\ C_2H_6 \longrightarrow$ 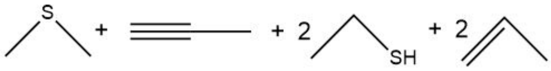

2-ethylthiophene – S7

CBH-0: $C_6H_8S + 9\ H_2 \longrightarrow 6\ CH_4 + H_2S$

CBH-1: $C_6H_8S + 6\ CH_4 + H_2S \longrightarrow 2\ CH_3SH + 2\ C_2H_4 + 3\ C_2H_6$

CBH-2: $C_6H_8S + 2\ CH_3SH + 2\ C_2H_4 + 2\ C_2H_6 \longrightarrow$ 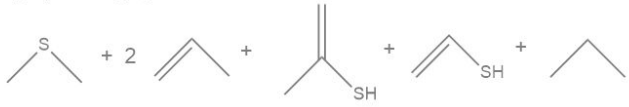

CBH-2: $C_6H_8S + 2\ CH_3SH + 2\ C_2H_4 + 3\ C_2H_6 \longrightarrow$ 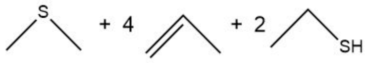

2-propylthiophene - S8

CBH-0: $C_7H_{10}S + 10\ H_2 \longrightarrow 7\ CH_4 + H_2S$

CBH-1: $C_7H_{10}S + 7\ CH_4 + H_2S \longrightarrow 2\ CH_3SH + 2\ C_2H_4 + 4\ C_2H_6$

CBH-2: $C_7H_{10}S + 2\ CH_3SH + 2\ C_2H_4 + 3\ C_2H_6 \longrightarrow$ 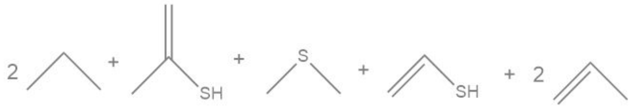

CBH-2: $C_7H_{10}S + 2\ CH_3SH + 2\ C_2H_4 + 4\ C_2H_6 \longrightarrow$ 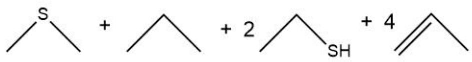

2-butylthiophene – S9

CBH-0: $C_8H_{12}S + 11\ H_2 \longrightarrow 8\ CH_4 + H_2S$

CBH-1: $C_8H_{12}S + 8\ CH_4 + H_2S \longrightarrow 2\ CH_3SH + 2\ C_2H_4 + 5\ C_2H_6$

CBH-2: $C_8H_{12}S + 2\ MeSH + 2\ C_2H_4 + 4\ C_2H_6 \longrightarrow$ 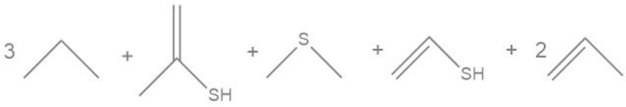

CBH-2: $C_8H_{12}S + 2\ CH_3SH + 2\ C_2H_4 + 5\ C_2H_6 \longrightarrow$ 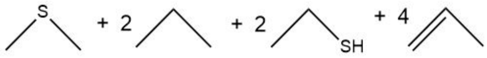

2-pentylthiophene – S10

CBH-0: $C_9H_{14}S$ + 12 H_2 ⟶ 9 CH_4 + H_2S

CBH-1: $C_9H_{14}S$ + 9 CH_4 + H_2S ⟶ 2 CH_3SH + 2 C_2H_4 + 6 C_2H_6

CBH-2: $C_9H_{14}S$ + 2 MeSH + 2 C_2H_4 + 5 C_2H_6 ⟶ 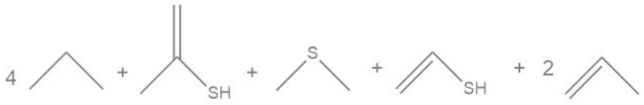

CBH-2: $C_9H_{14}S$ + 2 CH_3SH + 2 C_2H_4 + 6 C_2H_6 ⟶ 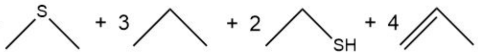

2-hexylthiophene – S11

CBH-0: $C_{10}H_{16}S$ + 13 H_2 ⟶ 10 CH_4 + H_2S

CBH-1: $C_{10}H_{16}S$ + 10 CH_4 + H_2S ⟶ 2 CH_3SH + 2 C_2H_4 + 7 C_2H_6

CBH-2: $C_{10}H_{16}S$ + 2 CH_3SH + 2 C_2H_4 + 6 C_2H_6 ⟶ 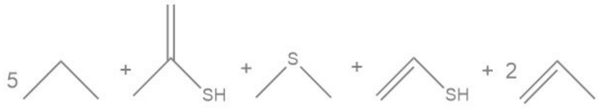

CBH-2: $C_{10}H_{16}S$ + 2 CH_3SH + 2 C_2H_4 + 7 C_2H_6 ⟶ 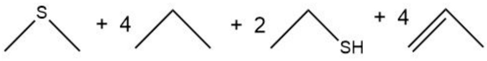

3-methylthiophene – S12

CBH-0: C_5H_6S + 8 H_2 ⟶ 5 CH_4 + H_2S

CBH-1: C_5H_6S + 5 CH_4 + H_2S ⟶ 2 CH_3SH + 2 C_2H_4 + 2 C_2H_6

CBH-2: C_5H_6S + 2 CH_3SH + 2 C_2H_4 + C_2H_6 ⟶ 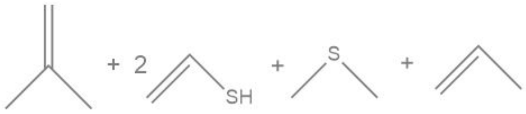

CBH-2: C_5H_6S + 2 CH_3SH + 2 C_2H_4 + 2 C_2H_6 ⟶ 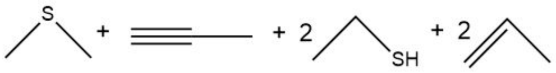

3-butylthiophene – S13

CBH-0: $C_8H_{12}S$ + 11 H_2 ⟶ 8 CH_4 + H_2S

CBH-1: $C_8H_{12}S$ + 8 CH_4 + H_2S ⟶ 2 CH_3SH + 2 C_2H_4 + 5 C_2H_6

CBH-2: $C_8H_{12}S$ + 2 CH_3SH + 2 C_2H_4 + 4 C_2H_6 ⟶ 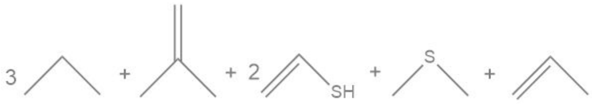

CBH-2: $C_8H_{12}S$ + 2 CH_3SH + 2 C_2H_4 + 5 C_2H_6 ⟶ 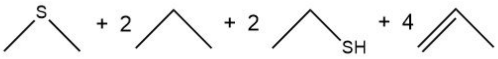

3-hexylthiophene – S14

CBH-0: $C_{10}H_{16}S$ + 13 H_2 ⟶ 10 CH_4 + H_2S

CBH-1: $C_{10}H_{16}S$ + 10 CH_4 + H_2S ⟶ 2 CH_3SH + 2 C_2H_4 + 7 C_2H_6

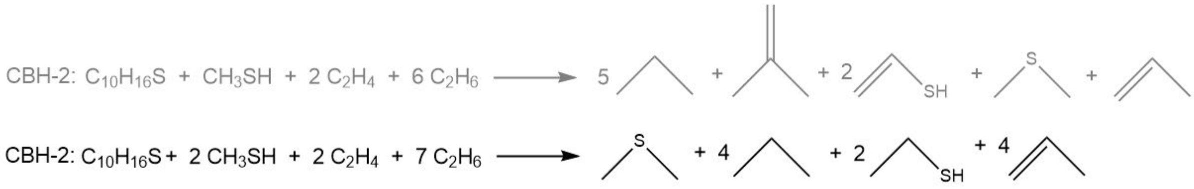

Figure S1. Zeroth, first and, second rung CBH reactions of alkyl-substituted thiophenes.
